# Striatal Toe: Too Harmless to Treat?

**DOI:** 10.3390/toxins17040168

**Published:** 2025-04-01

**Authors:** Wolfgang H. Jost, Emir Berberovic

**Affiliations:** 1 Parkinson-Klinik Ortenau, 77709 Wolfach, Germany; e.berberovic@parkinson-klinik.de; 2 Department of Neurology, University of Saarland, 66421 Homburg, Germany

**Keywords:** striatal toe, botulinum neurotoxin, Parkinson’s disease

## Abstract

A striatal toe is a misalignment of the hallux in dorsal flexion that frequently presents as a symptom of Parkinson’s disease and also atypical Parkinson syndromes. It can negatively impact patients during activities such as walking, putting on socks and shoes, and particularly while wearing shoes. It causes pain and thus induces a loss of quality of life. But, to date, we have few data on the topics of the prevalence, genesis, and therapy of striatal toe. Publications available on botulinum neurotoxin (BoNT) have demonstrated a positive effect in the treatment of striatal toe, although the current study data are also rather limited in this area. Commensurate approval studies have not yet been performed. We will introduce our contemporary data on therapy for striatal toe with BoNT and we will also discuss possible questions open for further study.

## 1. Introduction

Striatal toe is not an uncommon symptom, especially in patients with Parkinson’s disease (PD). It often leads to a relevant impairment of the patients’ quality of life. There is relatively little study data available on its causes and, above all, on treatment options.

The nomenclature is regrettably inconsistent. Striatal toe is a malposition of the hallux in dorsiflexion, without further evidence of functional damage to the cortico-spinal tract [1]. In addition, some studies indirectly also classify plantar flexion in the hallux as cases of striatal toe [2] and additional lateral flexion of the hallux is described as a severe form of this deformity [3]. This often occurs in combination with flexion of the remaining toes and equinovarus, which is also called striatal foot [4].

The symptom typically occurs in the context of PD, but can also be present in atypical Parkinson syndromes (PS) [2]. In addition, it has been described in the context of ischemic lesions in the area of the basal ganglia [5] and in primary dystonia [6].

Since the description of striatal toe is not uniform in all publications and there is often an overlap of symptoms with other causes of dorsiflexion of the hallux, a correct diagnosis as well as differentiation from dorsiflexion of the hallux of other genesis are essential.

Based on most publications, it is typical for a striatal toe (1) to occur in the context of PD or other neurodegenerative PS, (2) to persist with little dependence on or completely independent of dopaminergic stimulation (in On- and Off-phases), and (3) to often be accompanied by the remaining deformities of the same foot [1,4,7]. Striatal toe can be provoked in the early phase with plantar stimulation [1], while, later, this also occurs spontaneously, or the hallux remains in a fixed dorsiflexion with more or less limited active and passive mobility [4,7].

A difference between striatal toe and dystonia in the context of PD is the fact that, in striatal toe, there is usually a fixed dorsiflexion of the hallux, in contrast to dystonia, in which it is usually present intermittently in the context of fluctuations in dopaminergic stimulation (wearing-off or biphasic dystonia), it can be provoked by activation in the early phase, and is usually accompanied by pain [4]. In the initial phase, dystonia can be distinguished by fluctuations and provocation by activation, but, in the further course of untreated dystonia, it can lead to a fixed contracture. At this stage, it is difficult to distinguish it from striatal toe.

A distinction should also be made from Babinski’s sign, which is triggered by plantar stimulation and in which spreading of the remaining toes is typically observed, which is not the case with striatal toe and in which plantar flexion of the remaining toes is triggered [1]. Furthermore, the Babinski sign often occurs together with other signs of damage to the upper motor neuron.

Rheumatoid arthritis can also lead to a deformity of the hallux. However, in the case of arthritis, there is swelling and redness in the area of the toe, usually occurring bilaterally, and there are corresponding radiological changes, which is not the case with striatal toe [4].

The pathogenesis of striatal toe is currently not understood. Possible causes are in discussion, including striatal dopamine deficiency, a dystonic component, and fibrosis with altered plasticity and viscoelasticity of the local connective tissue [4]. A correlation with axial misalignment (e.g., camptocormia, anterocollis, or Pisa syndrome) is striking, which also occurs in the context of PD or PS, respectively, and the cause of which is also largely unclear and which cannot always be explained by dystonia.

Striatal deformities of the foot can lead to pain, impaired stance and gait stability, and the inability to wear shoes. With prolonged pressure lesions, ulceration and bone erosion can develop.

The hallux plays a decisive role in generating propulsive forces, weight bearing, foot clearance, and support of the foot in different gait phases [8]. A large part of a person´s weight is placed on the hallux when standing [9] and walking [10], so a loss of the function of the toe leads to a significant impairment of stance and gait.

## 2. Study Data

Given the overall limited study data on this topic, we have evaluated all publications available in PubMed since 1990 to the present regarding striatal toe, striatal foot, and up-going toe, as well as striatal deformities of the foot. We did not include publications that did not havedirect relevance to the topic, whose definition of striatal toe was too broad and imprecise, or in which the differentiation from other causes of up-going toe was not considered (Table 1). In addition, we searched for articles investigating the efficacy of BoNT in the treatment of lateral flexion of the hallux within the context of hallux valgus.

Overall, there are little study data on the frequency of striatal toe in PD (Table 2). In addition, the determination of its prevalence complicates the different definitions of the symptom in different studies, as well as possible misdiagnoses in the case of dorsiflexion of the hallux of other origins.

In a study by Winkler et al., more than 20% of patients with neurodegenerative PS were found to have striatal toe [1]. Here, a striatal toe was defined as an “apparent extensor plantar response, without fanning of the toes, in the absence of any other signs suggesting dysfunction of the cortico-spinal tract”. In the same study, the patients with striatal toe were more likely to have dyskinesia and more basal ganglia lesions were described on imaging.

In a retrospective study, Ashour et al. described striatal foot in 5.9% of 202 patients with neurodegenerative PS (PD, multiple system atrophy, and progressive supranuclear palsy) [2]. In this study, a striatal foot was defined as a fixed deformity (“may be partially overcome in early stages by voluntary or passive movement”) extension or flexion of the hallux, and equinovarus. The patients with a striatal deformity were younger and had an earlier onset of the disease than the patients without such a deformity. In addition, in 83% of the patients, the side of the striatal foot correlated with the side of the initial symptoms of PD.

Deformities of the extremities (hand and foot) are described by Jankovic and Tintner in up to 10% of untreated patients with advanced PD [11].

In a Mexican study, a striatal foot was observed in 1.2% of 416 patients with PD [12]. In this study, a striatal toe was defined as in the study by Winkler et al. [1].

**Table 2:** Frequency of striatal toe/striatal foot in Parkinson syndromes.

Study	Diagnosis	Patients (Total Number)	Striatal Toe and/or Striatal Foot (%)
Winkler et al. (2002) [1]	PD, MSA, PSP, DLB	62	20.97
Ashour et al. (2006) [2]	PD, MSA, PSP	202	5.94
Cervantes-Arriaga et al. (2016) [12]	PD	416	1.2

PD = Parkinson’s disease, MSA = multiple system atrophy, PSP = progressive supranuclear palsy, DLB: dementia with Lewy bodies.

There are currently little study data on the treatment of striatal toe in PD. The available material refers to the treatment of the striatal foot, or an “up-going toe”, in dystonia and after stroke with botulinum neurotoxin (BoNT).

In the only study we know of on the treatment of striatal foot in PD patients, by Lindholm et al. [13], therapy with incobotulinum toxin A with an individually adjusted dose demonstrated a standardized measured improvement in balance and gait, as well as a reduction in pain, cramps, and muscle tension. In all patients, BoNT was injected into the extensor hallucis longus muscle, but also, depending on the clinical findings, into the flexor digitorum longus, posterior tibialis muscle, and gastrocnemius muscle. After four weeks, the maximum effect was seen, which wore off after 16 weeks.

Giladi et al. reported a small series of patients (two with PD and one patient with generalized dystonia) with a “dystonic up-going toe” that impaired gait and led to pain [14]. The patients were treated with 50 to 70 U of onabotulinum toxin A in the extensor hallucis longus muscle and showed a subjective and objective improvement in symptoms of 80 to 90%. The therapy resulted in moderate weakness of the extensor hallucis longus muscle, but this did not affect the gait.

Yelnik et al. treated 11 patients with an “overactivity of the extensor hallucis longus muscle” in hemiplegia after a stroke with 66 to 100 U of onabotulinum toxin A in the extensor hallucis longus muscle [15]. On day 15, a regression of the overactivity of the extensor hallucis longus muscle was observed in 10 patients. During this time, a very good subjective effect on pain and difficulties with wearing shoes could be observed, and remained good or very good after three months in eight patients.

In a study by Kurtis et al. with eight patients with an up-going toe in dystonia or after stroke, higher doses of BoNT (type A or B) were injected [16]. Individual doses of BoNT from 40 to 160 U (onabotulinum toxin equivalent units) were administered every three months. Five of the patients had dystonia (one of which had off-dystonia in PD) and three had hemiparesis after stroke. A mean benefit of 62 ± 20% was achieved and the score of the Fahn–Marsden Dystonia Scale decreased by 1.8 ± 0.6. None of the patients reported adverse effects and the weakness of the extensor hallucis longus muscle remained without functional impairment.

Regarding the treatment of lateral flexion of the hallux in advanced striatal toe, there are no concrete data on treatment with BoNT. However, BoNT was used to treat hallux valgus in a placebo-controlled study by Wu et al. [17] and showed a reduction in misalignment and pain after injection into the adductor hallucis, flexor hallucis brevis, and extensor hallucis longus muscles. In another placebo-controlled study, the primary endpoint of pain reduction could not be reached after eight weeks, but the post hoc analysis showed a trend towards significantly more time in the “reduced pain state” with the higher dosage of 500 U abobotulinum toxin A after 12 weeks [18,19].

## 3. Practical Approach

**Figure 1:**
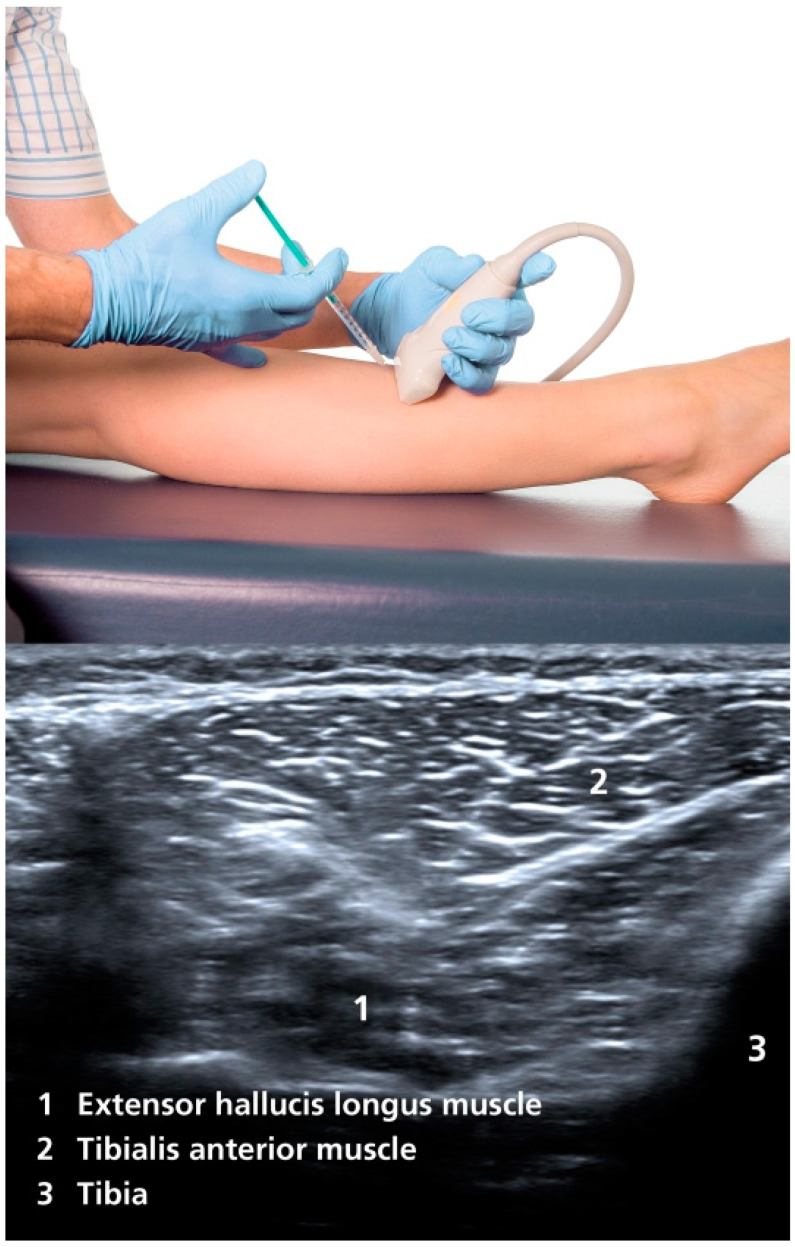
Injection site and ultrasound imaging of the extensor hallucis longus muscle [21].

By injecting BoNT into the extensor hallucis longus muscle, a reduction of the dorsiflexion of the hallux in striatal toe can be achieved. The injection should always be carried out under ultrasound control to avoid incorrect injection. The extensor hallucis longus muscle originates from the middle half of the fibula and the adjacent interosseous membrane, extends deeply between the anterior tibial muscle and the extensor digitorum longus muscle, and terminates at a tendon that attaches to the base of the terminal phalanx of the hallux [20]. The injection is made between the distal and middle third of the lower leg, along the lateral edge of the tibia [21]. At this height, the muscle can be visualized dorsal to the anterior tibial muscle and lateral to the tibia with ultrasound (Figure 1). This is often difficult to distinguish from the extensor digitorum longus muscle; therefore, it is helpful to activate the muscle (dorsiflexion of the hallux) in order to be able to better demarcate it. The usual dosage is initially 20 to 40 U of incobotulinum toxin A or onabotulinum toxin A, or 80 to 140 U of abobotulinum toxin A, although the dose can be titrated higher, depending on the therapeutic response (we usually start with a lower dose). The injection should be repeated and adjusted every three to four months. It should be noted that there is no official approval for the treatment of striatal toe with BoNT and that the therapy is a case-by-case decision.

## 4. Discussion

In the current studies, the prevalence of striatal toe is reported as a range between 1.2% and 20.97% in different cohorts (PD alone or combined with atypical PS). Due to the low number of patients in these studies and different cohorts, the global prevalence cannot be calculated. Based on our clinical experience, a relatively higher prevalence (greater than 20%) is to be assumed. Most patients are unaware of the deformity, and it is only with the progression of this state that everyday life becomes impaired. These patients usually are not able to establish a connection between the striatal toe and stance or gait instability. In the study by Lindholm et al. [13], an improvement in balance and gait was observed with BoNT therapy in patients with a striatal foot. However, the extent to which the treatment of the striatal toe influences this remains unclear. Further studies that explore the relationship between the striatal toe and stance and gait stability through standardized measurements would be useful. The use of higher doses of BoNT would be of interest, especially since no significant disadvantages have been observed in previous studies under higher doses. 

In addition, studies on the influence of striatal toe on the risk of falling in PD patients could thus further emphasize the value of early therapy of striatal toe.

On the other hand, a correlation between striatal toe and pain, as well as difficulties in wearing shoes, is frequently observed. Additionally, pain-related difficulty in walking accompanied by flexion of the remaining toes can also be observed in patients with a striatal foot.

## 5. Summary

Striatal toe is a relatively common symptom of PD and atypical PS, and can lead to impaired gait, pain, and wound formation in the toe area and thus to an impairment of the patient’s quality of life. Overall, however, there are little study data on the symptom and its very definition differs between individual studies, so a more precise prevalence cannot be clearly determined.

The advantage of this review lies in summarizing the existing studies and providing the reader with an overview of the problems arising from striatal toe, emphasizing its impact on foot functionality and the resulting limitations in the patient’s daily life. The need for therapy and the potential of treatment with BoNT are also discussed. Furthermore, this review highlights the need for further studies that would help in the treatment of patients with a striatal toe.

The weaknesses lie in the limited data available from studies with small sample sizes. The differing definitions of striatal toe in the existing studies also limit the possibility of a consistent evaluation of the available study data.

The striatal toe should be assessed in every patient with PD or an atypical PS and considered during the clinical examination to ensure an early diagnosis. BoNT therapy should be considered as soon as a disability caused by the striatal toe arises. Further studies are urgently needed to develop additional therapeutic approaches to better help affected patients.

## Figures and Tables

**Table 1:** Overview of the included and excluded articles.

Studies on “Striatal Toe”, “Striatal Foot”, “Up-Going Toe” or “Striatal Deformities of the Foot” Available on PubMed from 1990 to Present		
Included	10	
Excluded	9	
	No direct relevance to the topic	3
	Definition of striatal toe too broad and imprecise	4
	Differentiation from other causes of up-going toe not considered	6

## Data Availability

No new data were created or analyzed in this study. Data sharing is not applicable to this article.
